# Single-Element and MIMO Circularly Polarized Microstrip Antennas with Negligible Back Radiation for 5G Mid-Band Handsets

**DOI:** 10.3390/s22083067

**Published:** 2022-04-16

**Authors:** Falih M. Alnahwi, Yasir I. A. Al-Yasir, Chan Hwang See, Raed A. Abd-Alhameed

**Affiliations:** 1 Department of Electrical Engineering, College of Engineering, University of Basrah, Basrah 61001, Iraq; falih.mousa@uobasrah.edu.iq; 2 Faculty of Engineering and Informatics, University of Bradford, Bradford BD7 1DP, UK; r.a.a.abd@bradford.ac.uk; 3 School of Engineering and the Built Environment, Edinburgh Napier University, Edinburgh EH10 5DT, UK; c.see@napier.ac.uk

**Keywords:** microstrip antenna, circular polarization, axial ratio, reflection coefficient, mutual coupling

## Abstract

In this paper, single-element and MIMO microstrip antenna with two pairs of unequal slits is proposed as a circularly polarized antenna with negligible back radiation for 5G mid-band handsets. The unequal pairs of slits are engraved on the antenna patch to guarantee the presence of the circular polarization (CP). The proximity-coupled feeding technique is used to excite the proposed microstrip antenna in order to provide larger antenna −10 dB bandwidth which approaches 10.8% (3.48–3.87 GHz). A novel analysis technique is proposed in this paper that demonstrates the 3D axial ratio pattern in order to generate CP in the broadside direction without affecting the structure of the ground plane which ensures weak back radiation. The 3 dB axial ratio bandwidth (ARBW) is found to be equal to 4.1% extended along the range (3.58–3.73 GHz). To make the design more compatible with the 5G mid-band handsets, the 2 × 2 MIMO structure of the proposed antenna with reduced mutual coupling (less than −20 dB) is also presented in this work. The simulation and measured results are in good agreement, and both verify the CP characteristics and the weak back radiation of the proposed antenna.

## 1. Introduction

The main significance of the circular polarization (CP) substantially comes from its noticeable mitigation for the multipath interference and polarization mismatching [1,2]. These outstanding features attract the researchers’ attention to designing planar and non-planar antennas whose radiation is circularly polarized. In recent years, the utilization of CP has been oriented toward many wireless applications, such as some satellite applications, WiMax, WLAN, and 5G applications. Consequently, a circularly polarized antenna plays a vital role in these kinds of applications; therefore, it is necessary to seriously investigate some novel designs that guarantee CP at the broadside direction with compact size and suitable bandwidth coverage.

There have been many attempts to design antennas with multiband CP [3,4,5] and wideband CP [6,7,8] to cover as large a bandwidth as possible. However, these designs have significant back radiation that is not circularly polarized. Therefore, a major part of the radiation does not contribute to the CP communication. As a result, many researchers have directed their attention to the design of CP antennas with reduced back radiation to avoid the unnecessary linearly polarized back radiation in addition to the design of MIMO structures to accommodate the 5G requirements. In [9], the microstrip antenna is fed by a microstrip feed line to obtain narrow band CP with main lobe to back lobe level ratio equal to 4 dB. The main lobe to back lobe level ratio improved in [10] to 30 dB but with a very narrow fractional axial ratio bandwidth (ARBW) approaching less than 1.5%. The design in [11] also obtains low main lobe to back lobe level ratio but also with a narrow fractional ARBW that is equal to 2.14%. The ARBW bandwidth is enhanced to about 7% with 10 dB main lobe to back lobe level ratio using the complex and non-planar structures of the dielectric resonator antennas (DRA) with coaxial feeding technique [12,13]. The ARBW is widened to 3% with 16 dB main lobe to back lobe level ratio in [14] using a large-dimensions leaky-wave antenna. With the aid of coaxial feeding [15], the ARBW improved to 6% but with large back radiation whose main lobe to back lobe level ratio approaches 3 dB. In addition to the polarization diversity, when the circularly polarized wave reflects from any surface, it undergoes a reversal in the direction of the rotation [1]. As a result, the reflected wave never interferes with the incident wave. This advantage is very beneficial for mitigating the multipath effects. Therefore, it can be exploited for 5G and mobile communication applications which severely suffer from these kinds of interferences. In [16], a CP antenna is proposed with reconfigurable capability for switching between the left-hand and the right-hand CP for 5G mid-band applications. A wearable circularly polarized antenna for 5G and health care applications is proposed in [17] with the aid of metamaterial stacked structure. The CP beam steerable antenna array for 60 GHz 5G applications is presented in [18] by utilizing 8 × 8 Butler matrix. The CP antenna is also used for 5G smartphones [19,20] due to its mitigation for the multipath effects. All the aforementioned works tolerate either the ARBW or the back lobe level. Thus, it is necessary to design a planar antenna with low back lobe level, acceptable ARBW, and compact size.

This paper presents a circularly polarized microstrip antenna with two unequal pairs of slits for mid-band 5G applications. The proposed microstrip antenna is fully covered by the ground plane to avoid the undesired back radiation. In addition, the proximity-coupled feeding technique is selected to excite the proposed antenna to provide a broadband impedance matching. Based on the 3D axial ratio pattern, the direction of the CP is oriented toward the broadside of the antenna by controlling the width of each slit engraved on the antenna radiating patch. Since this novel analysis does not affect the structure of the ground plane, weak back radiation of the microstrip antenna is maintained. Moreover, a 2 × 2 MIMO structure is proposed in this work to accommodate the 5G handsets with minimized mutual coupling. The simulation and measured results are well agreed, and both show a good impedance bandwidth and acceptable ARBW. Furthermore, the results also show a broadside power pattern with negligible back radiation to avoid radiating in the undesired direction.

## 2. Antenna Structure

**Figure 1:**
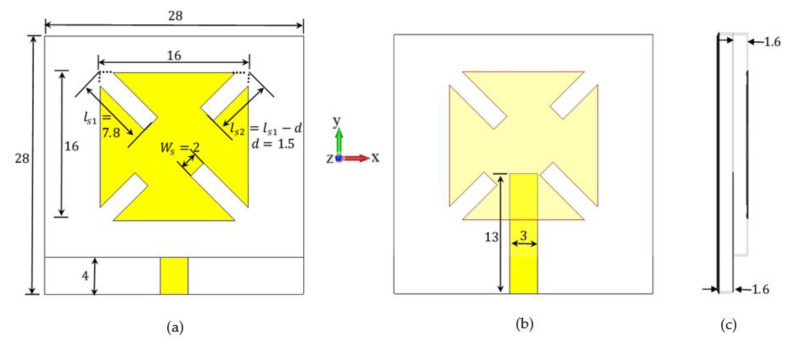
The structure of the proposed single-element CP antenna with two unequal pairs of slits with its optimized parameter values (**a**) top view, (**b**) the feed line structure, and (**c**) the side view. (All dimensions are in mm).

The structure of the proposed antenna is illustrated in Figure 1. The proposed design is a microstrip antenna that is fully covered by the ground plane. Moreover, the antenna consists of a double-layer structure with proximity-coupled feeding structure for broadening the microstrip antenna bandwidth [2]. The top view of the proposed antenna that is shown in Figure 1a consists of two unequal pairs of slits to provide two slightly separated resonant frequencies in order to attain the circular polarization (CP) criterion of the single feed structure [1]. If the length of the longer slit is equal to $l_{s1}$, then the length of the shorter slit is equal to $\left(l_{s2}=l_{s1}-d\right)$. The value of the difference in length (d) is optimized to obtain the suitable separation between the two resonant frequencies that results in a circular polarization. The width of each slit (Ws) is found to be a vital parameter that can control the bandwidth and the orientation of the CP. Figure 1b shows the structure and the dimensions of the 50 Ω feed line located in the second layer of the antenna. The antenna side view shown in Figure 1c is a composition of two 1.6 mm-thick FR4 dielectric material with dielectric constant equal to $\left(\varepsilon_{r}=4.3\right)$ and loss tangent equal to 0.025. The dimensions of the resulting antenna are 28 × 28 mm^2^.

## 3. Broadside CP Generation Mechanism

At first, it is worth mentioning that the simulation results of the proposed designs are obtained with the aid of CST Microwave Studio Simulation Suite manufactured by Dassault Systèmes UK, Coventry, United Kingdom.

**Figure 2:**
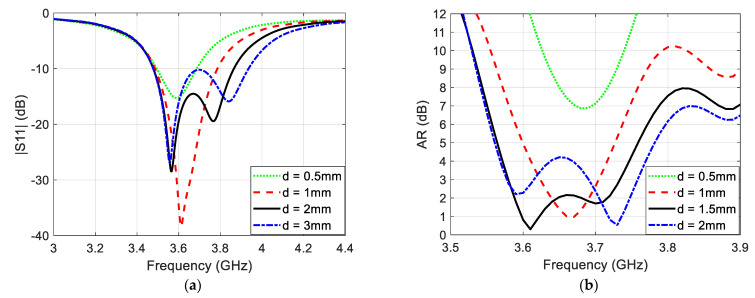
(**a**) Reflection coefficient and (**b**) broadside AR of the proposed microstrip antenna at Ws=2 mm and different values of d.

The basic criterion of generating the CP using single-feed structure is concluded by providing two perpendicular current paths with slightly different lengths in order to generate two adjacent resonant frequencies [1]. Moreover, engraving slits on the antenna patch electrically elongates the current path so that it noticeably minimizes the antenna dimensions [1]. This basic idea was achieved using probe feeding with narrow slots or slits which resulted in a very narrow-band circularly polarized radiation pattern in the broadside [1]. This basic concept initializes the design of the microstrip antenna shown in Figure 1, and then it is developed to improve the CP bandwidth in the broadside direction. Figure 2a,b illustrates the simulated reflection coefficient and broadside axial ratio (AR), respectively, of the proposed antenna for different values of slit length difference (d). It is clear that at d=0.5 mm, the difference in length between the two pairs of silts are so small that the separation between the two resonant frequencies is negligible. For this reason, there is no CP at this value of slit length difference. The same can be said for the case of d=1 mm but with little improvement in the AR value because the two resonant frequencies barely start to separate from each other. At d=1.5 mm, the separation between the two resonant frequencies becomes wider, so the 3 dB axial ration bandwidth (ARBW) has been improved to cover bandwidth of 150 MHz along the range (3.58–3.73 GHz). Increasing the value of (d) to 2 mm leads to a very wide frequency separation which results in noticeable loss in the CP bandwidth.

In fact, it is hard to obtain a broadside CP using the microstrip feeding or the proximity-coupled feeding. In this paper, it is noticed that these types of feeding can generate CP but not in the broadside direction. Therefore, one should find the key parameter that can justify the orientation of the CP toward the broadside direction. In the proposed design, the slit width ($W_{s}$) is found to be the vital key parameter that directly modifies the orientation of the CP since it can control the direction of the current path without a sensible effect on the resonant frequencies of the antenna. In addition, since there is no slit or slot engraved in the ground plane, the back radiation of the resulting antenna is still negligible.

**Figure 3:**
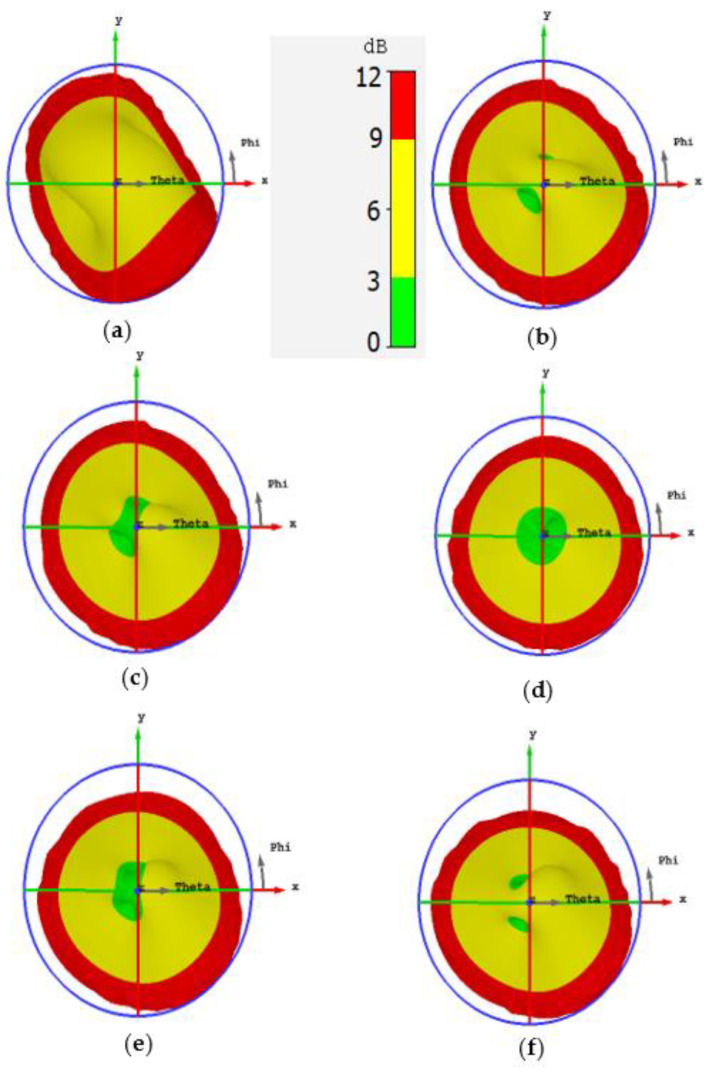
The axial ratio pattern of the proposed microstrip antenna for various values of slit width at 3.6 GHz using CST microwave Studio. (**a**) $W_{s}=0.5\;\mathrm{mm}$; (**b**) $W_{s}=1\;\mathrm{mm}$; (**c**) $W_{s}=1.5\;\mathrm{mm}$; (**d**) $W_{s}=2\;\mathrm{mm}$; (**e**) $W_{s}=2.5\;\mathrm{mm}$; (**f**) $W_{s}=3\;\mathrm{mm}$.

Figure 3 illustrates the 3D AR pattern of the proposed antenna for different values of slit width at 3.6 GHz. It is clear from Figure 3a that the CP is entirely absent in the broadside direction. However, by increasing the slit width to 1 mm, two circularly polarized regions (the green spots) start to appear as shown in Figure 3b. A slit width equal to 1.5 mm brings the two circularly polarized regions close to each other almost within the broadside orientation (see Figure 3c). The overlapping of the two circularly polarized regions in the broadside direction is completed at slit width equal to 2 mm as revealed in Figure 3d. The reason behind this overlapping is that increasing the slit width compels the electrical surface current of the antenna to concentrate in the central part of the antenna patch. As a result, the rotation of the electrical current is restricted in the central part of the patch which results in CP in the broadside direction.

It is known that the slit width has a minor effect on the location of the resonant frequencies because it does not contribute to elongating the current path. However, increasing the slit width to more than 2 mm makes the frequency shifting sensible, and this leads to modifying the separation between the two resonant frequencies. Consequently, the condition of the CP may be broken for large values of slit width. This can be verified by taking a look at Figure 3e where the two CP regions start to detach from each other, and Figure 3f shows how the CP is lost at the broadside when the slit width is equal to 3 mm.

**Figure 4:**
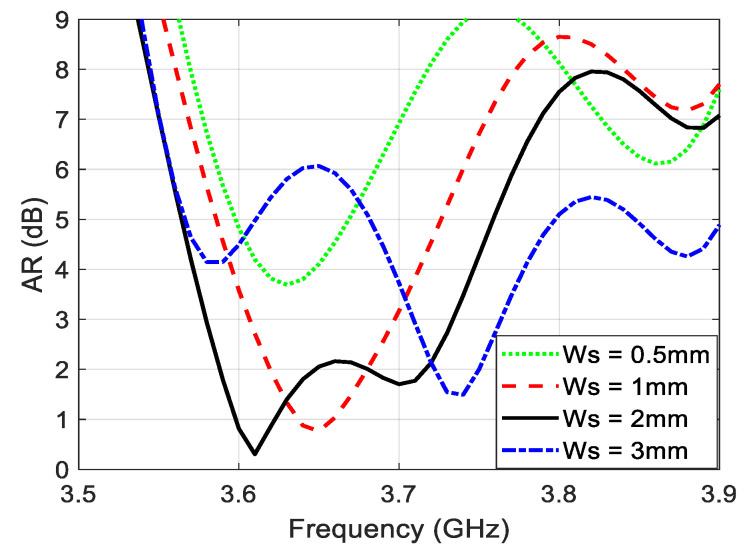
Broadside AR of the proposed microstrip antenna of slits at d=1.5 mm and different values of Ws.

**Figure 5:**
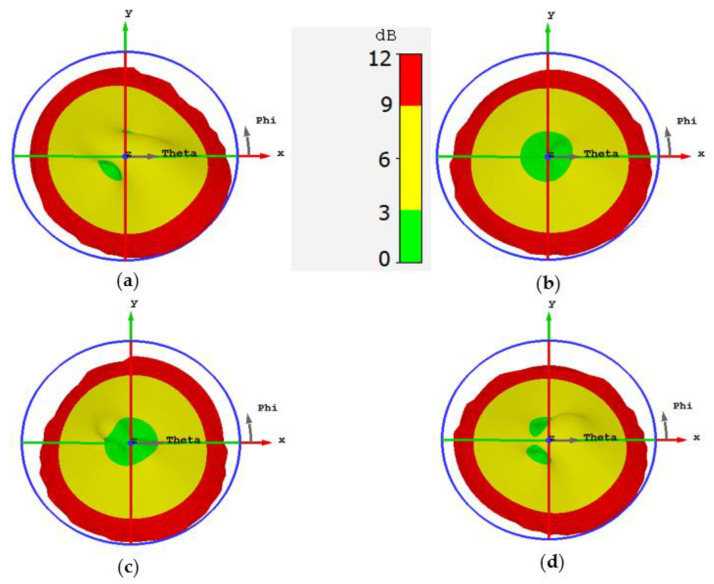
The AR pattern of the proposed antenna at various frequencies. (**a**) f=3.57 GHz; (**b**) f=3.6 GHz; (**c**) f=3.7 GHz; (**d**) f=3.74 GHz.

The bandwidth of the CP is improved in this design thanks to the presence of the two CP regions. The broadside CP is generated by overlapping two CP regions, so the CP cannot easily be lost because that requires the two CP regions to be completely detached from each other. Figure 4 illustrates the AR as a function of frequency which shows a CP bandwidth equal to 150 MHz. Figure 5 demonstrates the AR pattern for different frequencies. Figure 5a shows no CP in the broadside because the frequency is out of the CP bandwidth. Figure 5b,c exhibit a good overlapping for the CP regions in the broadside direction. The broadside CP is lost in Figure 5d where the frequency is not within the bandwidth of the CP.

**Figure 6:**
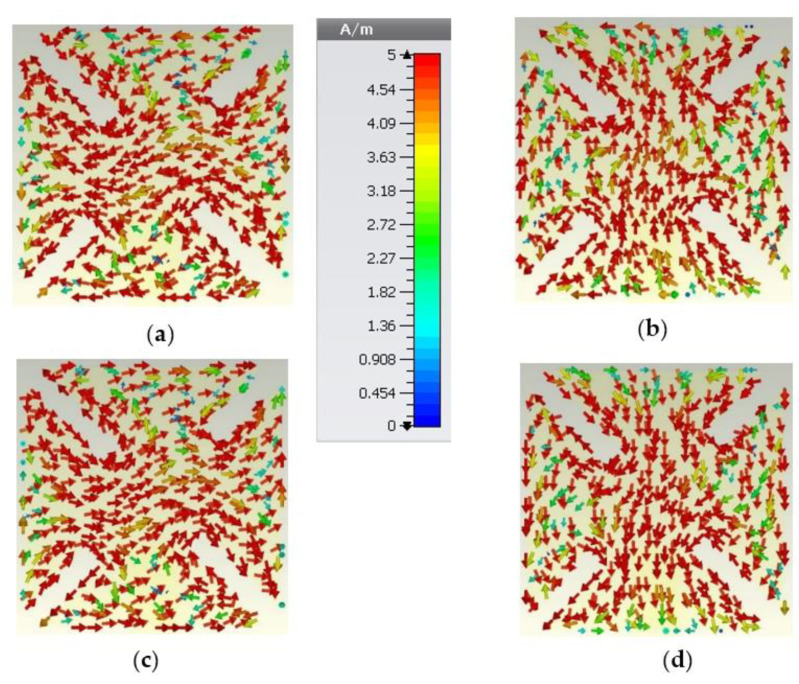
The current distribution on the radiating patch of the proposed antenna at different time instants at f=3.6 GHz. (**a**) $\omega t=0^{{}^{\circ}}$; (**b**)$\;\omega t=90^{{}^{\circ}}$; (**c**) $\omega t=180^{{}^{\circ}}$; (**d**) $\omega t=270^{{}^{\circ}}$.

Finally, to verify the presence of the CP within the broadside direction, the current distribution at the antenna patch is demonstrated in Figure 6 for different time instants at 3.6 GHz. The rotation of the current can clearly be seen in the central part of the radiating patch, and this confirms the presence of left-hand circular polarization (LHCP) in the broadside direction. If right-hand circular polarization (RHCP) is required, the positions of the longer and shorter pairs of slits should be swapped.

## 4. Results

This section presents the simulation and the measured results of the single-element and MIMO structures of the proposed microstrip antenna. The measurements were acquired at University of Bradford/Faculty of Engineering and Informatics using Agilent N5242A vector network analyser.

### 4.1. Single-Element Structure

**Figure 7:**
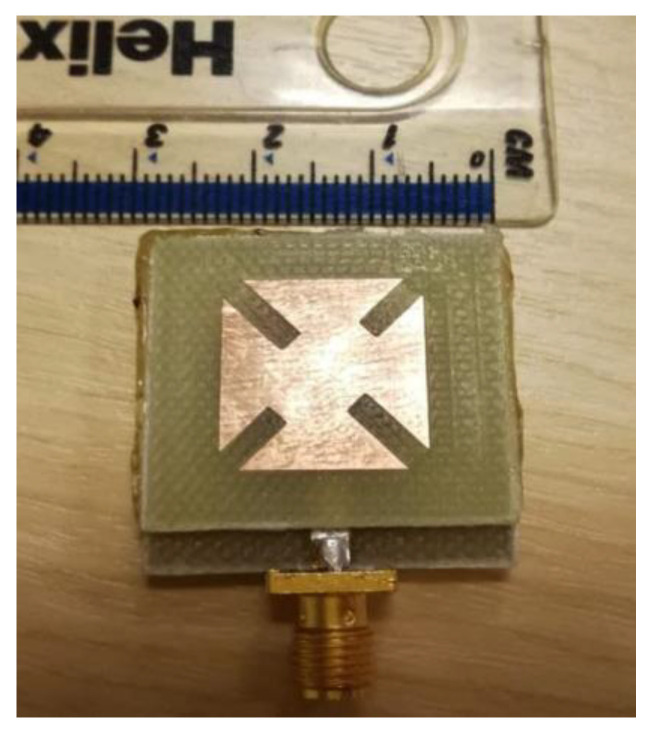
Prototype of the proposed single-element circularly polarized microstrip antenna.

**Figure 8:**
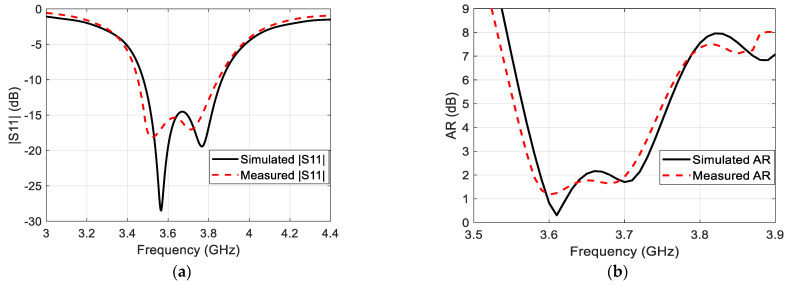
Simulation and measured (**a**) reflection coefficient and (**b**) AR of the proposed single-element circularly polarized microstrip antenna.

Figure 7 illustrates the prototype of the proposed microstrip antenna. The top and bottom layers were joined using cyanoacrylate adhesive material. The simulated and measured reflection coefficients are demonstrated in Figure 8a, while the simulated and measured AR at the broadside direction of the antenna as a function of frequency is exhibited in Figure 8b. The simulated impedance bandwidth of the proposed antenna is found to be equal to 10.8% along the range (3.48–3.87 GHz), while the measured impedance bandwidth is equal to 10.9% along the range (3.46–3.86 GHz). On the other hand, the simulated ARBW is equal to 4.1% extended along the range (3.58–3.73 GHz), whereas the measured ARBW is found to be equal to 4.12% along the range (3.57–3.72 GHz).

**Figure 9:**
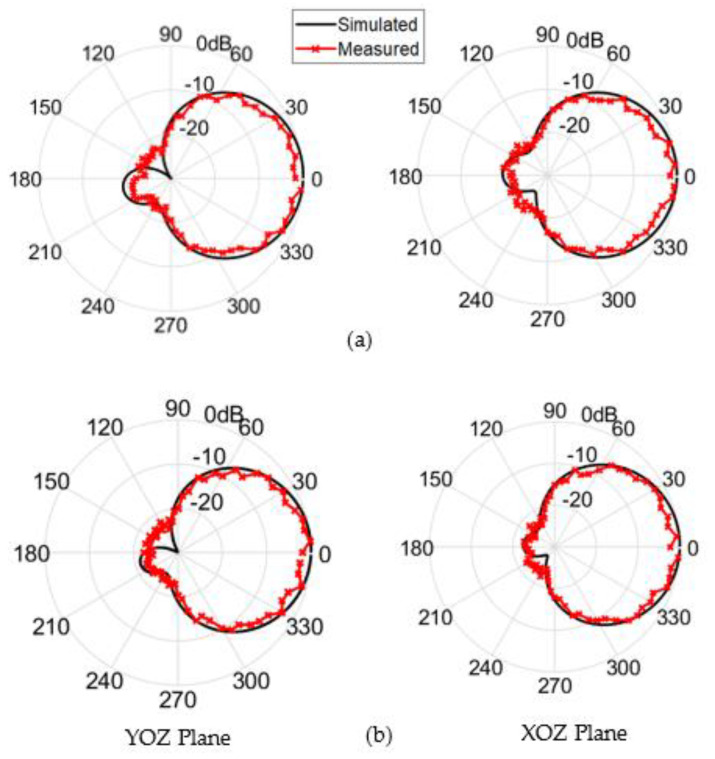
Simulation and measured normalized power patterns of the proposed single-element circularly polarized microstrip antenna at (**a**) 3.6 GHz and (**b**) 3.7 GHz.

**Figure 10:**
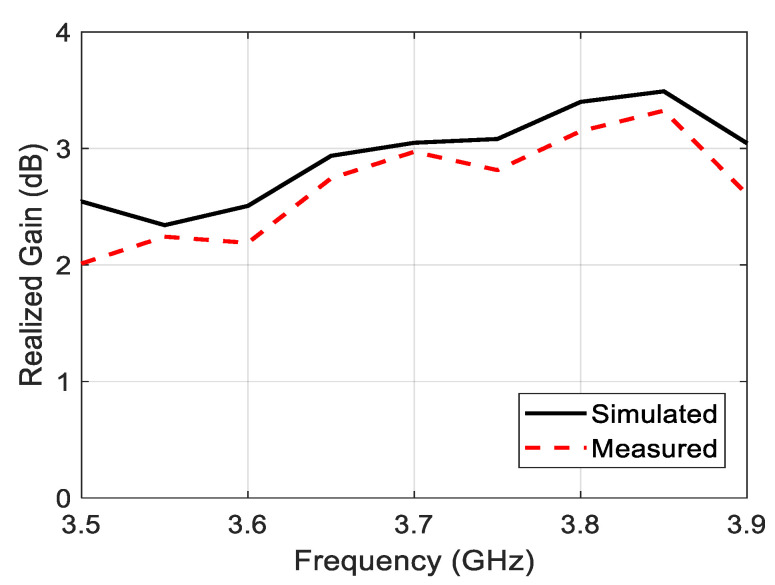
Simulation and measured maximum realized gain of the proposed single-element circularly polarized microstrip antenna.

The simulated and the measured normalized power patterns at the YOZ and XOZ planes of the proposed antenna at 3.6 GHz and 3.7 GHz are revealed in Figure 9. By taking a look at these normalized power patterns, one can easily deduce that the antenna has a broadside radiation with a subtle amount of back radiation with main lobe to back lobe level ratio that approaches values larger than 20 dB. Unlike the omnidirectional pattern, the small amount of back radiation is very important in the 5G handsets to protect the consumer’s head from the radiation of these devices. Figure 10 illustrates the simulated and the measured maximum realized gain over the entire operating band of the antenna. This figure shows a very acceptable gain value along the frequency range of interest. The deviation between the simulated and the measured gain is attributed to many factors, such as the reflections coming from the surrounding equipment inside the chamber, the irregular distribution of the dielectric constant with the frequency, and the losses caused by the imperfect soldering and the adhesive material used to stick the two layers of the antenna.

### 4.2. MIMO Structure

**Figure 11:**
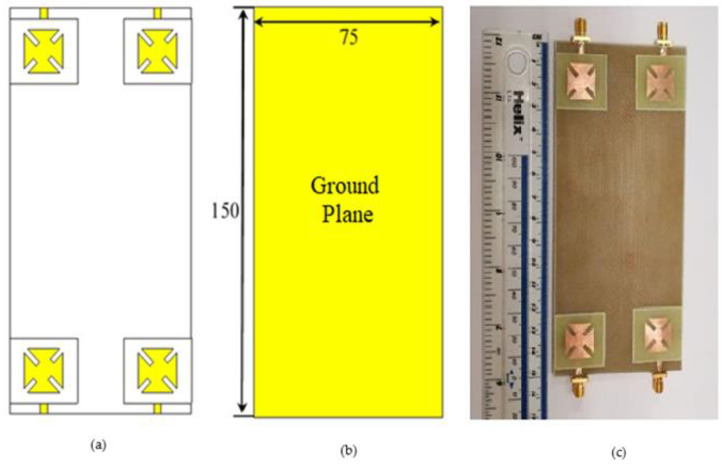
The proposed MIMO structure (**a**) top view of the antenna, (**b**) back view of the antenna, and (**c**) the prototype of the proposed MIMO antenna. (All dimensions are in mm).

This work exploits the previous single-element antenna structure to design 2 × 2 MIMO structures as shown in Figure 11. Figure 11a,b represent the front and the back views of the proposed MIMO antenna, while Figure 11c shows the prototype of the proposed design. The proposed MIMO structure is etched on FR4 dielectric substrate with the standard cellphone dimensions (150×75 mm). The antenna elements are arranged at the corners of the substrate in such a way that all the antennas give LHCP. It is important to notice that all the dimensions of the single antenna are kept as they are.

**Figure 12:**
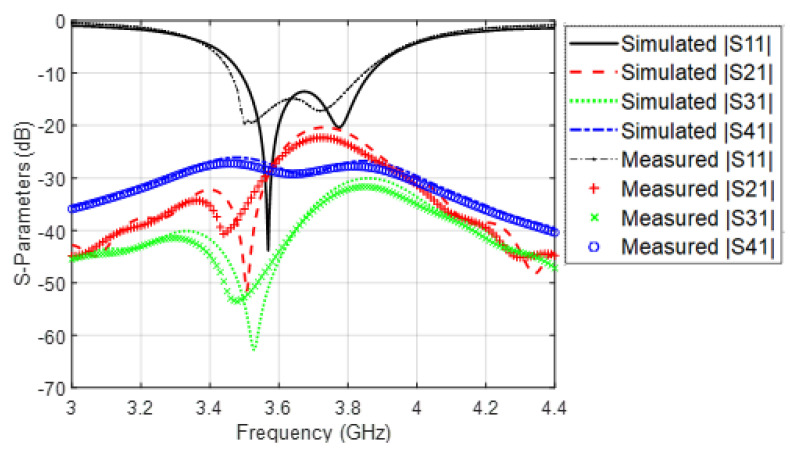
Simulated and measured s-parameters of the proposed MIMO structure.

The mutual coupling between the four antennas can be demonstrated with the aid of the transmission coefficients between the antennas. Unlike the non-symmetrical MIMO structure which requires the forward and the backward transmission coefficients [21,22], the symmetrical MIMO structures requires just the forward transmission coefficient in describing the mutual coupling between the antennas. Figure 12 shows the simulated s-parameters of the MIMO antenna, whereas Figure 12 illustrates the measured s-parameters of the proposed MIMO antenna. Since the MIMO antenna have transmission coefficients less than −20 dB, the isolation between the antennas is almost perfect without the need for adding isolation elements because it is less than the margin of −15 dB [9,23,24]. The Envelop Correlation Coefficient (ECC) between every two antenna elements (say 1 and 2) is given by [25]:

(1) $$ECC=\frac{{\left|S_{11}{}^{*}S_{12}+S_{22}S_{21}{}^{*}\right|}^{2}}{\left(1-\left({\left|S_{11}\right|}^{2}+{\left|S_{21}\right|}^{2}\right)\right)\left(1-\left({\left|S_{22}\right|}^{2}+{\left|S_{12}\right|}^{2}\right)\right)}$$

**Figure 13:**
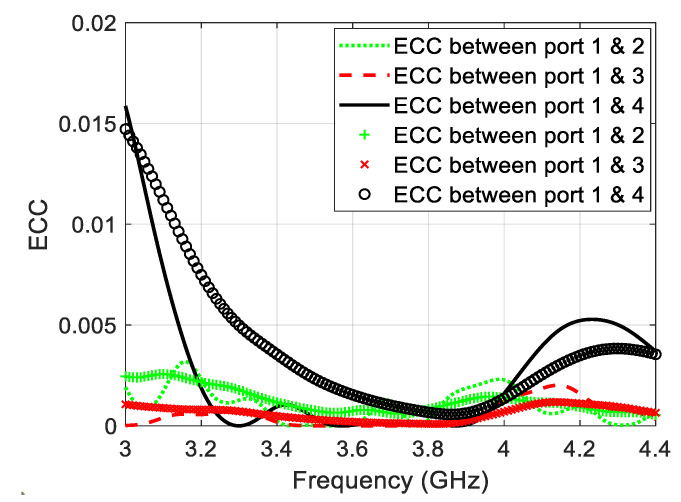
Simulated and measured ECC of the proposed MIMO structure.

Figure 13 illustrate the simulated and the measured ECC of the proposed MIMO antenna structures as a function of frequency. In general, the ECC has values less than 0.02, and this is another important evidence about the excellent isolation between the antenna elements of the proposed design. The deviation between the simulated and measured results in Figure 12 and Figure 13 comes from the imperfect soldering of the SMA connector, the fabrication imperfection, as well as the adhesive material which is not considered in the simulation process.

As a matter of comparison, Table 1 gives a comparison between the proposed design and some other important works in terms of the impedance bandwidth, ARBW, and main lobe to back lobe level ratio. It is clear from this table that the proposed antenna provides an incredible balance between the width of the ARBW and the level of the back radiation.

## 5. Conclusions

A microstrip antenna with proximity-coupled feeding and two pairs of unequal slits for 5G mid-band applications has successfully been proposed in this paper. Based on the 3D axial ratio pattern, a novel analysis technique is used to orient the CP toward the broadside of the antenna without distorting the ground plane in order to keep the back radiation as low as possible. The simulated impedance bandwidth of the antenna is equal to 10.8% along the range (3.48–3.87 GHz), while the measured impedance bandwidth is equal to 10.9% along the range (3.46–3.86 GHz). On the other hand, the simulated ARBW is equal to 4.1% occupying the range (3.58–3.73 GHz), whereas the measured ARBW extends along the range (3.57–3.72 GHz) with fractional value equal to 4.12%. The main lobe to back lobe level ratio of the proposed antenna is found to be 20 dB, which guarantees weak radiation toward the undesired radiation. A 2 × 2 MIMO structure has also been designed in this work with mutual coupling less than −20 dB and ECC less than 0.02.

## Figures and Tables

**Table 1:** Comparison between the proposed design with some important designs related to some other important designs.

Ref.	Impedance BW %	ARBW %	Main Lobe to Back Lobe Level Ratio (dB)
[9]	13.8%	4.5%	4 dB
[10]	7%	1.5%	30 dB
[11]	4.2%	2.14%	20 dB
[14]	11.1%	3%	16 dB
[15]	10.5%	6%	3 dB
Proposed	10.9%	4.12%	20 dB

## Data Availability

Not applicable.
